# Knowledge, attitudes, and practices regarding cystic echinococcosis and sheep herding in Peru: a mixed-methods approach

**DOI:** 10.1186/s12917-017-1130-4

**Published:** 2017-07-06

**Authors:** Veronika Merino, Christopher M. Westgard, Angela M. Bayer, Patricia J. García

**Affiliations:** 1 Kuskaya: An Interdisciplinary Training Program for Innovation in Global Health, School of Public Health, Universidad Peruana Cayetano Heredia, Av. Honorio Delgado 431 San Martin de Porres, Lima, Peru; 2 Kuskaya: An Interdisciplinary Training Program for Innovation in Global Health, School of Public Health, University of Washington, Seattle, USA; 3 Division of Infectious Diseases, David Geffen School of Medicine, University of California, Los Angeles, California, Los Angeles USA; 4 Unit of Epidemiology, STD and HIV, School of Public Health and Administration, Universidad Peruana Cayetano Heredia, Lima, Peru

**Keywords:** Echinococcosis, *Echinococcus granulosus*, Zoonoses, Surveys, Peru

## Abstract

**Background:**

The parasitic disease, cystic echinococcosis (CE), is prevalent in low-income, livestock-raising communities and 2000 new people will be diagnosed this year in South America alone. The disease usually passes from livestock to dogs to humans, making it a zoonotic disease and part of the One Health Initiative. Control of CE has been infamously difficult; no endemic areas of South America have succeeded in maintaining sustainable eradication of the parasite.

For the current study, we aimed to gain a better understanding of the knowledge, attitudes, and practices of rural sheep farmers and other community leaders regarding their sheep herding practices and perspectives about a control program for CE. We also hope to identify potential barriers and opportunities that could occur in a control program.

The authors conducted Knowledge, Attitude and Practices (KAP) surveys and semi-structured interviews in rural communities in the highlands of Peru. The KAP surveys were administered to 51 local shepherds, and the semi-structured interviews were administered to 40 individuals, including shepherds, community leaders, and health care providers.

**Results:**

We found that the shepherds already deworm their sheep at a median of 2 times per year (*N* = 49, range 2–4) and have a mean willingness-to-pay of U.S. $ 0.60 for dog dewormer medication (*N* = 20, range = 0.00- $2.00 USD). We were not able to learn the deworming agent or agents that were being used, for neither sheep nor dogs. Additionally, 90% of shepherds slaughter their own sheep (*N* = 49). We also learned that the main barriers to an effective control program include: lack of education about the cause and control options for CE, accessibility to the distant communities and sparse grazing pastures, and a lack of economic incentive.

**Conclusions:**

Findings suggest it may be feasible to develop an effective CE control program which can be used to create an improved protocol to control CE in the region.

**Electronic supplementary material:**

The online version of this article (doi:10.1186/s12917-017-1130-4) contains supplementary material, which is available to authorized users.

## Background

Cystic echinococcosis (CE) is one of the most prevalent zoonotic diseases in South America [1]. It is caused by an infection of the larvae of the parasite *Echinococcus granulosus.* Humans can become infected by ingesting the eggs excreted by a dog or any other canid (definite hosts); however, humans cannot transmit the disease (accidental host). The eggs ingested by humans and/or intermediate hosts (livestock, such as sheep, cattle, horses, etc.) reach the gastrointestinal tract where they hatch, freeing oncospheres that are transported through lymphatic and cardiovascular circulation to various organs, usually the liver or lungs, where they will lodge and slowly form cysts, causing CE [2, 3].

CE is considered by the World Health Organization (WHO) to be a neglected disease [4], and it remains a persistent problem in areas of low socio-economic status and livestock production. The present study was set in the Central and Southern Andes of Peru, regions where sheep-herding communities are found. While some of these communities also raise bovines and South American camelids, and CE can be present in these species as well, the study focused in sheep-herding communities given that the prevalence of the disease has been reported to be higher in sheep raising regions.

This disease causes a substantial burden in highly endemic areas of South America, including Peru [5]. The WHO Informal Working Group on Cystic Echinococcosis Surveillance, Prevention and Control [6] estimates that CE results in 1 to 3 million Disability-Adjusted Life Years (DALYs) per year. The disease increases vulnerability for the poorest populations due to medical costs and loss of human and livestock productivity [7, 8]. Some studies have shown that CE can result in a 10% decrease in productivity of infected animals, lowering quality of meat, production of fiber and milk, and number of surviving offspring [9].

For effective control of *E. granulosus,* it is necessary to stop the parasite’s development at different stages of its life cycle [10]. There are several control strategies that have been studied and have proven to be successful in different scenarios [10, 11]; some of the control strategies that have been studied are: dog deworming, sheep deworming and sheep vaccination [12–16]. Mathematical modelling has shown that the most effective intervention was a combination of vaccinating sheep and dog deworming treatment [10].

There have been several attempts at controlling CE in endemic countries around the world; however, most endemic areas, like Latin America, have not achieved sustainable control. Potential barriers have been discussed in various control studies, including unsustainable financial and political support, inaccessible roads and communities, poor evaluation efforts, lack of education and supplies in the control authority, and ineffective infrastructure to administer frequent dog deworming [1, 13]. Most of these studies include administrative difficulties as one of the main barriers, but lack in-depth information regarding the perspectives and daily practices of intervention recipients.

Therefore, the aim of the present study is to explore: the knowledge, attitudes and practices of shepherds regarding CE; the shepherds’ willingness-to-pay for certain key control strategies; and the perspectives of community leaders, health center representatives, and local municipality employees regarding CE. With a better understanding of the perspectives of diverse stakeholders we hope to identify and target barriers to the implementation of an effective control program.

## Methods

### Study design

This was a descriptive, cross-sectional, mixed methods study. The qualitative semi-structured interviews provide a cross reference to responses given in the KAP survey. Triangulation between the qualitative and quantitative data helped generate a comparative analysis, increase the credibility of results and limit misinterpretations [17].

### Study area

The study was conducted from November to December 2014, in the central and southern highlands of Peru, in the provinces of Junin, Huancavelica, Puno, and Cusco. Villages from 14 districts with sheep husbandry practices were sampled. The villages are located at high altitudes, normally varying between 3200 to 4100 m above sea level. Transportation to these villages takes from 1 to 6 h by car from the capital of each department. The villages within each region were chosen using purposive sampling to ensure that different areas of the region were represented in our sample population.

### Preliminary work

The research team conducted a stakeholder analysis to identify the actors at the national level (in Lima) that have knowledge regarding CE and its control. The authors received assistance from representatives from the Pan American Health Organization (PAHO) and Universidad Peruana Cayetano Heredia (UPCH) to coordinate with other political and academic experts. Representatives from the Ministry of Health, Ministry of Agriculture, and Ministry of Housing, Construction and Sanitation – Programa Nacional TAMBO (PNT) [18] assisted in introducing the research team to contacts within the study communities and facilitating logistical information. The PNT provides, in isolated high poverty areas of Peru, buildings designed to host representatives of National, Regional and Local institutions to facilitate the access to this often-neglected population and ensure they can be reached and covered by different services and programs. These buildings include dormitories; media-equipped offices and meeting/conference rooms; and a storage room stocked with emergency equipment and supplies.

### Data collection and analysis methods

#### Quantitative methodology

The quantitative component was carried out with community members within sheep raising communities. The research team utilized purposive sampling within the communities by traveling through the streets or pastures searching for individuals that owned sheep and/or owned dogs and approaching them to inquire about participation in the study.

All participating community members completed a Knowledge, Attitudes and Practices (KAP) survey. The KAP survey was administered, with the interviewer reading the questions in Spanish, without presenting the response options so that participants could provide the answers they deemed most appropriate. If the participant provided an answer that was not listed as an option, the new answer was included in the questionnaire under ‘Other’. The KAP survey was divided into three sections and included questions regarding the shepherds’ knowledge and experience with CE in animals and humans; demographics of their animals; and medication and slaughter practices for their animals. The KAP survey included 47 questions and took approximately 20 to 30 min to complete.

A randomly selected sub-group of participating shepherds was asked to complete a willingness-to-pay survey, which was presented following the KAP survey and a short description of the disease and of each of the three control strategies considered in the study. The shepherds were asked how much they would be willing to pay for the medication for each control strategy. The three control strategies presented were: (1) Praziquantel to deworm dogs every 45 days, (2) Oxfendazole to deworm sheep every three months, and (3) EG95 vaccine to vaccinate sheep three times in their first year of life. The shepherds were asked how much they would pay per dose. The interviewer started by asking if they would pay 20 Soles (U.S. $6.35), which is a very high price, considering the average shepherd’s economy (U.S. $ 100 per month, as stated in the interviews). If the shepherd declined that price, the interviewer proceeded to present lower and lower prices until the shepherd agreed on the price he would be willing to pay. Often the latter process was not necessary since after declining the high price, the shepherds would state the price they were willing to pay.

The data from the KAP surveys were entered in a database in Microsoft Excel® and reviewed for errors by another member of the research team. Data was analyzed using EpiInfo version 3.5.4 (Epidata Association; Odense, Denmark). First, descriptive data analysis was run, which included calculating frequency of responses, mean, and median. Then, univariate logistic regression models were applied to analyze correlations between the community members’ experience with CE, and the shepherds’ practices that would contribute to effective CE prevention. The authors wanted to see if previous exposure to information regarding CE had contributed to a positive change in practices. Prevalence ratios (PR) and their corresponding 95% Confidence Intervals, along with *p* values from the 2-tailed Fisher’s exact test, are provided for each regression.

#### Qualitative methodology

The qualitative component was carried out with community members including: shepherds; community leaders, health center representatives; and personnel from local municipalities. The qualitative component also utilized purposive sampling to seek out relevant participants. For non-shepherds, a few key actors were identified in the villages and in department capitals and they were asked to provide introductions to others that could offer relevant information. A semi-structured guide was used to interview participants in greater depth about their perspectives regarding the barriers to CE control programs in their communities. The interview began by asking participants what they and others in their community think about CE control programs, including difficulties they may face for each control program alternative. The interviews also engaged participants about the three potential control strategies (dog deworming, sheep deworming, and sheep vaccination) to learn about their associated barriers. The conversation was audio recorded with prior written consent, and later transcribed.

For the qualitative data analysis, the study team identified a set of sub-themes from the responses to generate an initial codebook. These codes were applied to two transcripts (one from shepherds, one from non-shepherds) to standardize the coding process. The resulting modified codebook and transcripts were entered into Dedoose (SocioCultural Research Consultants; Manhattan Beach, CA). Next, the team coded the remaining transcripts. Finally, they explored similarities and differences in perspectives of participants within and across participants.

## Results

### Quantitative

#### Cystic echinococcosis -relevant knowledge, Attitudes and practices among shepherds

Fifty-one (51) participants, individuals that either owned sheep and/or owned dogs, completed the KAP survey (the results of the KAP survey can be found in Additional file 1). The demographic information of the KAP participants, as well as information regarding their sheep and dogs and their practices with these animals, is displayed in Table 1. Of the interview participants (51), 49 owned sheep, with a median of 24 sheep per shepherd. Only 12% of shepherds have veterinary supervision of their sheep and 90% slaughter their own sheep. 41 out of 51 participants (80%) owned dogs, with a median of 2 dogs per shepherd.

**Table 1:** Characteristics of Knowledge, Attitudes and Practices (KAP) survey shepherd participants and information regarding their ownership of sheep and dogs and their practices with these animals, Peru, 2014

	Number (%)	Median (Range)
CHARACTERISTICS OF KAP SURVEY PARTICIPANTS, *N* = 51		
Gender	-	-
Male	30 (59)	-
Female	21 (41)	-
Age	-	45.5 (20–75)
CHARACTERISTICS OF SHEEP OWNED BY PARTICIPANTS		
Number of sheep owned	-	24 (2–417)
Sheep receive veterinarian supervision	6 (12)	-
Participants slaughter own livestock	44 (90)	-
Location of slaughter	-	-
Only in backyard	48 (98)	-
Backyard and Slaughterhouse	4 (10)	-
Backyard and Common area	1 (2)	-
Use of meat from sheep	-	-
Personal consumption	48 (98)	-
Sell meat in market	34 (69)	-
Does not use it (Sell sheep alive)	20 (41)	-
Age of sheep at slaughter or sale (years)	-	1.5 (0.5–5)
Maximum age of sheep (years)	-	10
CHARACTERISTICS OF DOGS OWNED BY PARTICIPANTS		
Owns a dog	41 (80)	-
Number of dogs owned	-	2 (1–6)

We found that 98% of the shepherds currently deworm their sheep, 60% of them to treat *Fasciola hepatica* (common liver fluke), while the rest deworm against diverse intestinal parasites and ectoparasites, at a median of 2 times per year, and at a median price of $0.38 per dose of dewormer. About 69% of the shepherds that deworm their sheep administer the medicine themselves or with a community member and 31% use a vet/technician. Regarding sheep vaccination, we learned that the shepherds referred to vaccines as anything injectable. Therefore, we were not able to differentiate between the use of vaccines and other injections like antibiotics or vitamins. 53% of shepherds provide injections at a median of 2 times per year and at a median price of $0.32 per injection. Less than half (39%) of shepherds that provide injectable medicine to their livestock administer the injections themselves, and 16% use a vet/technician. We also learned that most of the shepherds have seen cysts in their sheep’s viscera (89%). The shepherds indicated that they often bury (73%) or burn (35%) the viscera, if it is infected with cysts.

Of the participants who declared to own dogs, 73% currently deworm them, and at a median of 2 times per year. We were not able to learn the deworming agent or agents that were being used. More than half of the dog-owners feed sheep viscera to their dogs (56%). 83% of them indicated that they feed the viscera cooked (58%), and 15% of dog owners said that they do not feed them viscera if it has cysts.

Of all the shepherds surveyed, only 33% indicated they had heard of CE. When asked how the disease is transmitted to humans, only 4% correctly mentioned that it is transmitted through infected dog feces. 15% incorrectly responded that the disease is contracted by eating infected meat or viscera. After the participants learned about CE and its health consequences in humans, 35% of them stated that they knew someone that had contracted the disease (35%); of those infected people, 94% needed surgery.

#### Willingness-to-pay for cystic echinococcosis control strategies among shepherds

The Willingness-to-pay survey was administered to 22 individuals. The following number of participants responded to the three willingness-to-pay questions regarding three potential control strategies: dog dewormer (*n* = 19), sheep dewormer (*n* = 17), sheep vaccine (*n* = 20). The number of shepherds that answered each question of the *Willingness-To-Pay* survey varied because some were not able or did not agree to answer one or two of them. The results display the average the shepherd is willing to pay for one dose of the required medication to control the spread of CE in U.S. Dollars (3.15 Peruvian Soles = 1 USD; at the time of the study). The responses for how much they would be willing to pay were higher than the responses in the KAP survey regarding how much they currently pay for dewormer and vaccines for their sheep (U.S. $ 0.56 vs U.S. $ 0.38, and U.S. $ 0.70 vs U.S. $ 0.32, respectively). The averages for willingness-to-pay for all three medications are substantially lower in Puno than the other two provinces﻿ (See Table 2).

**Table 2:** Willingness-To-Pay for CE control strategies among Shepherds, Peru, 2014

AVERAGE WILLINGNESS-TO-PAY	CUSCO (U.S.$/dose)	PUNO (U.S.$/dose)	JUNIN (U.S.$/dose)	AVERAGE (U.S.$/dose)
Dog Dewormer	0.71	0.18	1.04	0.60
Sheep Dewormer	0.71	0.15	0.61	0.56
Sheep Vaccine	0.84	0.14	1.01	0.71

#### Association between cystic echinococcosis knowledge and experiences and cystic echinococcosis-relevant practices among shepherds

The relationships between knowledge of CE were regressed against the shepherds’ practices that would have an influence reducing CE. The results are displayed in Table 3. The only significant correlation is between having previously participated in a control program for CE and deworming their dogs 3 or more times per year (*p* = 0.02). The other correlations between knowledge of CE and CE-related practices were not significant.

**Table 3:** Results for Univariate Logistic Regression

	Deworms sheep ≥2 times per year	Deworms dogs ≥3 times per year	Feeds infected viscera to dogs
	Prevalence Ratios (95% CI). *p*-value		
Participated in CE^a^ control program	0.58 (0.23–1.45), *p* = 0.24	0.49 (0.21–1.16). *p* = 0.02^b^	0.94 (0.13–6.87), *p* = 1.00
Heard of CE^a^	0.82 (0.49–1.38), *p* = 0.54	0.92 (0.70–1.38), *p* = 1.00	0.83 (0.17–4.01), *p* = 1.00
Knew someone with CE^a^	1.18 (0.75–1.85), *p* = 0.55	0.76 (0.52–1.12), *p* = 0.22	0.30 (0.04–2.33), *p* = 0.37

^a^ *CE* Cystic echinococcosis

^b^significant at confidence of 95%

### Qualitative

Semi-structured interviews were held with 40 participants. The population included 19 shepherds; 10 community leaders, who also owned sheep; 5 health care providers from local Health Centers, including doctors, medical technicians and a nurse; and 6 Ministry of Housing, Construction and Sanitation – TAMBO National Program representatives. The quotes used to present these results can be found in Additional file 2.

#### Knowledge related to cystic echinococcosis

We found that most of the interviewees, including health care providers, did not have a clear notion of what CE is, nor how it is transmitted. Some interviewees thought that CE was transmitted through contact with cat hair, while others were convinced that CE was caused by the ingestion of meat from infected animals. Although some of them were aware that dogs are involved in the transmission of CE, they did not fully understand that livestock and humans are infected by ingesting *E. granulosus* excreted by dogs.

In regards of presence of CE in their community, some of the interviewees confirmed that they were familiar with some positive cases within their community. Some of these known cases were patients that had gone through surgery after symptoms became severe, and some other patients had passed away.

#### Barriers for a cystic echinococcosis control program

When shepherds and community leaders were asked about their perspective on a control program for CE, we obtained answers that reflected some of the most important barriers for a successful program, including: understanding the benefits of the program, their willingness to participate in a program, and difficulties accessing the population. Rural populations will only participate in a new program if they can see and experience immediate benefits. When they were asked about participating, some replied that currently one of the greatest incentive to participate in health programs and/or campaigns, like vaccination for children, is the presence of *‘Programa Juntos´,* a Peruvian Government cash transfer program to aid poor populations, financed by the Ministry of Social Inclusion and Development that places conditions for funding, such as maintaining children good school attendance, children vaccines up to date, etc.

There are some areas where pilot programs have been present, and have exposed the community to information about CE. However, several communities were omitted from pilot programs and other social initiatives due to their inaccessibility. Some of these villages do not have roads and it could take up to 10 h on foot to reach them. Interviewees from such populations stated that they feel like they have been neglected.

We found that for a significant number of shepherds, lack of knowledge and awareness about CE is an important barrier. For this population, education and training are immensely important, and are key factors to an effective and sustainable control program. They believe that continuous training and informative campaigns for shepherds would aid the fight against CE and other zoonotic diseases in livestock. Some community members consider that education must be conducted using strategies specifically designed for the target population. These strategies need to reflect their culture, be accessible and engaging. They recognize that raising livestock is their main source of income and that keeping their animals free of disease will translate into better economy.

Another interesting finding was that shepherds and other community members suggested having people from the community actively involved, and that they would prefer a member of their own community, rather than an ¨outsider¨ to act as a promoter, distributing preventive medicine. Some of the reasons they presented include the fact that they would be more familiar with their livestock, costumes and routine, and would be more responsible and involved with his or her community’s interests.

#### Perspectives on cystic echinococcosis control strategies

The implementation of dog deworming is a challenging procedure because sheep dogs are working dogs, and behave very differently than domestic dogs in urban areas. Shepherds acknowledge that sheep dogs are more difficult to handle and to medicate on a regular basis. In addition, local government representatives consider this option less viable, since they perceive that communities do not have “*a set routine, not even for themselves. They don’t go or take their kids to the health center. It is less likely that they would do it for their animals.”*

When asked about sheep vaccination interventions, interviewees presented different reactions. Some believe that sheep vaccination could be valuable, but were concerned about the difficulties in successfully tracing all the sheep from all the shepherds. Other shepherds were in complete agreement of implementing a vaccination intervention, since it would prevent the disease in animals, and therefore in humans as well. Additionally, people had different perspectives about the effect of the vaccination on the quality and flavor of the meat. Some shepherds recognized the potential benefit of producing better quality meat from disease-free animals; on the other hand, others were worried about the possibility of the vaccine altering the flavor of the meat, following their experience with their potato crops after artificial fertilizers were introduced.

Shepherds and community leaders were also inquired about deworming of sheep. Their responses show that deworming could be a potential strategy if the medication is long-lasting and low-cost, but challenges would include perceptions about investing in sheep that will soon be slaughtered. Shepherds also showed concern about the drug’s withdrawal period, its efficacy, and frequency of dosage.

#### Willingness to pay for cystic echinococcosis control strategies

Some shepherds indicated that if the medicine were provided for free by a program, they would have less confidence in the control program. They mentioned that if the drugs were distributed for free, they would be afraid that the medication would not be safe for their animals, that it might be expired or even toxic. A community leader said: *“Most of the people will want to make sure that the medicine they are getting is real, and of good quality, or at least the same quality as the medicine they regularly buy themselves. If they can make sure of that, they will accept it.”* Additionally, paying for treatment might increase compliance, since it is less likely that shepherds will not use something they already have paid for.

While shepherds stated that they will be willing to pay for intervention strategies, some ministries representatives, such as a PNT representative reported that in impoverished areas, having to pay could be a potential barrier: *“In Huancavelica, in the isolated areas, like the estancias, people only make a living from the sale of their livestock. In a month, they possibly sell one or two sheep, obtaining a monthly income of 200 to 300 Soles [U.S. $ 63 – U.S. $95]. If they need to invest an amount close to 10% of their monthly income, it is very unlikely that they will agree to it.”*

## Discussion

Our findings highlight the difficulties faced by a control initiative in poor, hard to reach regions, but also identify several opportunities. Even though CE can develop in most livestock, the study focused on sheep, because the highest prevalence of CE in humans is found in populations that raise sheep [19]. The study found that shepherds know very little regarding CE and that they deworm their sheep and dogs already to control intestinal parasites, but the frequency and the drugs they are currently using are not necessary effective against *E. granulosus*. However, they are willing to improve their deworming practices if they receive education about the benefits. The lack of awareness and understanding of the disease, and the low profit margins shepherds earn from their livestock could be some of the reason why control has been so ineffective. The intervention strategies to control CE that have been effective in wealthy countries have not been effective at achieving long-term control of the disease in low income countries [11]. In Peru, between 2002 and 2013, there were 33,838 human CE cases reported. The province of Pasco has the highest prevalence of CE, reporting 108 cases per 100,000 persons, followed by Huancavelica (43/100,000) [20]. These two regions are the hardest to reach; the poorest and several of the comments in these regions noted the lack of government assistance they receive.

In fact, the barriers to an effective control program in the highlands of Peru are likely greater than any other region in South America. The communities are in high-altitude, difficult to reach regions of the Andes, shepherds are some of the poorest populations in Peru, and the education system is often very low-quality in remote regions. Also, the inability of programs to effectively control CE could be attributed to the fact that chronic diseases with low fatality rates that are limited to poor rural areas are particularly “unattractive” to researchers and funders who depend on relatively immediate results. CE shares this situation with other communicable diseases, such as neurocysticercosis and Buruli disease [21]. Effective control is hindered because surveillance is difficult in animals and humans, infection is asymptomatic in dogs, clinical signs in livestock are not easily perceived by shepherds and the disease can take 5–10 years to be symptomatic in humans [4].

Many of our findings are contradictory to beliefs held by some policy makers in the country’s capital of Lima. During the preliminary phase of the study we held meetings with policymakers, some of whom perceived that people in rural areas will not accept CE-related intervention programs. Our results show that most of the people we interviewed would be willing to adopt a new program if they are provided with sufficient education about benefits and risks. Also, as previously mentioned, they are already deworming (although not in the right frequency and/or dosage recomeme﻿nded to control Echinococcosis) and report being willing to pay for deworming medicine. People in the areas we visited, along with previous studies, consider education to be the key to any sustainable program [2]. Shepherds showed great interest in the topic of CE once they recognized the potential risks for their families’ health and the negative effect on their income.

Many policy makers and experts were not aware that shepherds already deworm their livestock and dogs. Since 73% of dog owners already deworm their dogs, and 98% of shepherds already deworm their sheep to control intestinal parasites, a control program would only need to explain and encourage increased frequency and dosage for their sheep and assure the correct drugs to treat Echinococcosis are used, while complementing the control of other parasites. Currently shepherds deworm their sheep before and after rain season (December to March), therefore any new deworming protocol should expand on the same deworming schedule [11].

The price for preventative medication was one of the main barriers we heard from shepherds and community leaders for adopting CE prevention strategies. Although, they are willing to pay for interventions, they are reluctant to increase the expenses needed to raise their sheep, specifically new medications that are unknown to them and are unsure of the efficacy. The study’s *willingness-to-pay* analysis found that the median willingness-to-pay amount for preventive medication was well below the market price of the drugs in some provinces and for certain control strategies, such as dog dewormer in Junin and Cusco. In poorer provinces, like Puno, the willingness-to-pay amount was significantly less than the market price for all control medication options. This aligns with the fact that Puno is the most impoverished of the three departments [22]. In poorer provinces like this, there would need to be substantial financial support. In regions with higher willingness-to-pay for control strategies, it should be relatively easily to implement a program, with appropriate education and logistical support.

Creating the required behavior change by shepherds to halt the spread of CE is another issue to address [15]. If the shepherds stopped feeding their dogs the sheep viscera, the spread of CE would come to a halt. A previous study in Peru showed that 83% of shepherds feed their dogs infected viscera [23]. Although 73% of shepherds indicated that they bury the infected viscera in our study, it is highly unlikely this occurs, due to social desirability; and when it does occur it is likely that they are not burying the viscera deep enough to prevent the dogs from digging it up and eating it. The effort that it would take to bury viscera deeper than a dog would dig, in the rocky ground of the Andes, is tremendous. Performing this act, every time a shepherd slaughters a sheep, is unreasonable. However, the shepherds still indicate that they do so. Also, because we found that 90% of shepherds slaughter their own sheep, we cannot rely on slaughterhouses to handle the disposal of the infected viscera. Behavior change must occur at the household level. Therefore, a better disposal strategy for sheep viscera must be developed.

35% of shepherds answered that they knew someone that had suffered from CE, and only 6% of the shepherds identified the correct form of disease transmission. Many of the shepherds were aware that CE is present in their communities, but did not know how it got there. Most of them think that it is due to ingesting infected sheep viscera. Most think that to prevent CE, they should offer sheep viscera to their dogs instead of to their families, inadvertently completing the biological cycle of *E. granulosus*.

Another important barrier for a control program is the difficult access to communities [10]. Usually, shepherding communities are in districts far from the province capital, and the shepherds spend many weeks at a time away in higher lands where better pastures can be found. These areas are between 1 and 10 h away on foot from their communities, and are hardly accessible by motor vehicles. This creates a burden for the shepherds to obtain preventive medicine from their communities.

Some shepherds said they are not willing to administer new medicine to their animals if they do not see any immediate benefit. Neither the dogs nor the sheep show evident clinical signs when infected with *E. granulosus*, so it is difficult to encourage compliance from owners to deworm their animals, especially if they are asked to do so frequently. Also, deworming dogs is very challenging. Handling sheepdogs is much more difficult than handling pet dogs, and it is difficult to monitor and ensure that dogs are getting the medicine they need in the frequency and dose required.

One of the limitations of the present study is related to data collection. It involved only 51 participants for the quantitative interviews and 40 participants for the qualitative interviews. For the study population to be more representative, it would have benefitted from a larger number of participants for the KAP surveys. The research team was not able to survey more shepherds due to limited accessibility and great distance to most sheep grazing fields and the fact that rural sheep-raising communities are highly dispersed. Another important limitation in this study is the potential bias on the willingness-to-pay values, due to that they were mostly based on responses from shepherds that already deworm their animals.

## Conclusion

The findings of this study illustrate that any effective and sustainable efforts to control CE require addressing the barriers perceived by the shepherds and to identify opportunities. The main barriers identified by this study include: lack of education about the cause and control options for CE; accessibility to the distant communities and sparse grazing pastures; complications in coordinating the administration of preventative medicine; and lack of economic incentives for CE control.

The opportunities found in this study are: most shepherds already deworm their sheep at a median of 2 times per year, slaughter their sheep at 1.5 years old or younger, and deworm their dogs at least twice a year; willingness to pay, at least a small amount, for interventions; and the acceptance of community workers to distribute preventive medicine and health messages. The findings can be used to create an improved, expanded protocol to control CE in the region.

As proposed in previous studies, education on Echinococcosis and its economic and public health impact on humans and animals, will support the behavior change needed to halt transmission of the parasite [24].

## Additional files

Additional file 1: Knowledge, Attitudes and Practices survey data. XLXS (44.9 KB)

Additional file 2: Qualitative. Semi-Structure interview quotes. XLXS (30.1 KB)

## Abbreviations

CE: Cystic echinococcosis
DALYs: Disability-Adjusted Life Years
KAP: Knowledge Attitudes and Practices
PNT: Programa Nacional TAMBO
PR: Prevalence ratios (PR)
WHO: World Health Organization
